# Predicting Outcomes in Frail Older Community-Dwellers in Western Australia: Results from the Community Assessment of Risk Screening and Treatment Strategies (CARTS) Programme

**DOI:** 10.3390/healthcare12131339

**Published:** 2024-07-05

**Authors:** Roger M. Clarnette, Ivan Kostov, Jill P. Ryan, Anton Svendrovski, D. William Molloy, Rónán O’Caoimh

**Affiliations:** 1 Medical School, University of Western Australia, Crawley, WA 6009, Australia; roger.clarnette@health.wa.gov.au (R.M.C.); ivan_kostov@baxter.com (I.K.); 2 Department of Nursing, Fiona Stanley Fremantle Hospital, 11 Robin Warren Drive, Murdoch, WA 6150, Australia; jillian.ryan@health.wa.gov.au; 3 UZIK Consulting Inc., 86 Gerrard St E, Unit 12D, Toronto, ON M5B 2J1, Canada; info@statshelp.ca; 4 Centre for Gerontology and Rehabilitation, University College Cork, St Finbarr’s Hospital, Douglas Road, T12 XH60 Cork, Ireland; w.molloy@ucc.ie; 5 Department of Geriatric Medicine, Mercy University Hospital, Grenville Place, T12 WE28 Cork, Ireland

**Keywords:** frailty, older adults, community dwelling, risk prediction, adverse outcome, prevalence, Aged Care Assessment Team, Comprehensive Geriatric Assessment

## Abstract

Understanding risk factors for frailty, functional decline and incidence of adverse healthcare outcomes amongst community-dwelling older adults is important to plan population-level health and social care services. We examined variables associated with one-year risk of institutionalisation, hospitalisation and death among patients assessed in their own home by a community-based Aged Care Assessment Team (ACAT) in Western Australia. Frailty and risk were measured using the Clinical Frailty Scale (CFS) and Risk Instrument for Screening in the Community (RISC), respectively. Predictive accuracy was measured from the area under the curve (AUC). Data from 417 patients, median 82 ± 10 years, were included. At 12-month follow-up, 22.5% (n = 94) were institutionalised, 44.6% (n = 186) were hospitalised at least once and 9.8% (n = 41) had died. Frailty was common, median CFS score 6/9 ± 1, and significantly associated with institutionalisation (*p* = 0.001), hospitalisation (*p* = 0.007) and death (*p* < 0.001). Impaired activities of daily living (ADL) measured on the RISC had moderate correlation with admission to long-term care (r = 0.51) and significantly predicted institutionalisation (*p* < 0.001) and death (*p* = 0.01). The RISC had the highest accuracy for institutionalisation (AUC 0.76). The CFS and RISC had fair to good accuracy for mortality (AUC of 0.69 and 0.74, respectively), but neither accurately predicted hospitalisation. Home assessment of community-dwelling older patients by an ACAT in Western Australia revealed high levels of frailty, ADL impairment and incident adverse outcomes, suggesting that anticipatory care planning is imperative for these patients.

## 1. Introduction

Functional decline and frailty are common among community-dwelling older adults [1,2] and influence the risk of adverse health-related events such as mortality [3]. If the risk of such outcomes can be determined, then allocation of medical resources can be planned more efficiently to mitigate this and support older adults to age in place in the community. Assessment utilising a variety of different risk-prediction models is increasingly being used in the community to triage patients [4,5]. Risk describes the amount of potential harm that can occur in a set period of time due to a specific event or series of events and is the product of the probability that harm will occur and the magnitude of its severity [6]. Rational decision making in healthcare requires reliable and valid quantitative ways of expressing risk that balance the potential costs and benefits of different management strategies [7].

Multiple factors including cognitive impairment, depression, medical comorbidities, low levels of physical activity and social isolation are associated with an increased risk of adverse healthcare outcomes among community-dwelling older adults [8]. Many but not all of these predispose people to the development of frailty [9]. These factors can be grouped into three main categories or domains: mental health including cognition, activities of daily living (ADLs) and medical conditions and comorbidities [10]. The ability of each individual’s caregiver network and social supports to manage the person’s care deficits also affects their level of risk [10]. Inadequate social or support networks contribute to poor healthcare outcomes, particularly risk of institutionalisation [11].

A variety of methods have been used in an attempt to identify community-dwelling older adults at risk. Many short risk-prediction instruments focus on the identification of frailty [12], an age-associated vulnerability to adverse outcomes [13], acting as short screens or surrogates for Comprehensive Geriatric Assessment (CGA) [14]. Non-targeted community screening is less efficient, suggesting that two-step assessment comprising rapid screening with risk-prediction or frailty instruments followed by triage and appropriate management of high-risk individuals may be most effective [15,16]. Although the stratification of risk scores using these instruments is associated with clinically meaningful gradients of adverse outcomes, most risk-prediction models have relatively poor predictive ability [4,5]. This is especially true at an individual level and may relate to a failure to incorporate important personalised social and demographic data into these assessments [4].

To date, most assessments of frail older adults occur in healthcare facilities, usually outpatient clinics in acute care. Clinicians that visit patients in their home are often in the best position to screen and assess older adults to deliver connected and integrated care based on CGA [15,17]. Nurses in particular play a key role in all areas of healthcare delivery in the community, including assessment of care needs, and can be trained to deliver specific interventions ranging from psychosocial strategies to interventions for chronic medical conditions [18] in the home environment. In some countries, patients with chronic illnesses such as dementia are more likely to be attended by nurses than other healthcare professionals [19]. Studies have found high levels of frailty-related risk factors among patients under nurse follow-up in the community [10,20,21]. Despite this, little is known about the pattern of risk factors, frailty prevalence and risk of adverse outcomes among older adults attended at home by community-based Aged Care Assessment Teams (ACATs) in Australia [22].

This study presents findings from the Community Assessment of Risk Screening and Treatment Strategies (CARTS) programme in Western Australia [23]. The purpose was first to establish the prevalence of risk factors for frailty and functional decline in a selected sample of community-dwelling older adults in Western Australia, second to identify factors associated with perceived risk of adverse outcomes in this population, third to investigate their distribution according to the severity of that perceived risk using a new risk stratification model, the Risk Instrument for Screening in the Community (RISC) [10], and fourth to determine the correlation of risk assessment with future risk of adverse outcomes.

## 2. Materials and Methods

### 2.1. The Outcome Measures

The CARTS programme was first developed in Ireland and has since been used in several countries around the world [24]. It uses bespoke instruments to measure risk of adverse outcomes comprising a brief screening instrument (the RISC) and a more detailed targeted CGA called the Community Assessment of Risk Instrument (CARI) [23]. The RISC was developed at University College Cork (UCC), Ireland, and first validated in 2015 [10]. The research team worked with public health nurses (PHN) in Ireland to develop the tool. This instrument was developed to measure risk of three specific adverse outcomes, institutionalisation, hospitalisation and death [10], in community-dwelling older adults who were receiving or had applied for community services. PHNs are community-based specialised nurses, known in the United Kingdom as district nurses and by other names in other countries. They are geographically based and work with individuals, families and communities to screen and monitor conditions and diseases. Following development of the tool, a cohort of PHNs undertook training on using the tool in practice. PHNs then used the RISC on older patients in their caseload to measure risk.

The RISC is a quick and easy instrument that identifies individuals at risk of the three adverse events (institutionalisation, hospitalisation and death) based on the presence or absence of subjective risk using a risk matrix (i.e., the combination of expected impact and expected severity) in relation to three health domains: mental state, ADLs (functional status in everyday activities) and medical state. Then after noting the patients’ living arrangements, the ability of the caregiver network to manage or negate/neutralize the concern is noted and the risk of each domain is graded from 1 (low) to 5 (high) [10]. The RISC can be used to screen large numbers of older persons quickly and efficiently. Health professionals using hypothetical case scenarios previously assessed the RISC for reliability; the study showed excellent internal consistency and inter-rater reliability [25]. Research has shown that it is an acceptable and valid instrument in a variety of languages and cultures [24,25,26].

Individuals who score medium–high on the RISC are then assessed in more detail using the CARI [23]. The CARI provides a more comprehensive risk assessment for the same three adverse outcomes. The instrument collects demographic data (age, educational level and living arrangements), details of home supports, caregiver burden, medical history, medications and hospital attendances in the preceding year. Taking this information into account, it records the presence and magnitude (low, medium, high) of concern within and across the same three domains, mental state, ADLs and medical state, but in more detail, highlighting specific deficits called issues, e.g., anxiety or depression, difficulty managing shopping and environmental or socioeconomic issues. In summary, there are seven items within the mental state domain, fifteen items within the ADL domain and nine items within the medical state domain. Based upon the caregiver networks’ ability to manage each domain, an overall *Global Risk Score* is then attributed to each adverse outcome. The *Global Risk Score* uses a five-point Likert scale based on a risk matrix, ranging from lower to higher, that is, one (minimal impact and rare event) to five (extreme impact and certain event).

### 2.2. Data Collection

The Department of Community and Geriatric Medicine (DCGM) at Fremantle Hospital and Health Service in Western Australia provides a comprehensive assessment service to older patients who live in the southern suburbs of the greater Perth metropolitan area in Western Australia. Clinical services include inpatient rehabilitation, outpatient assessment, day therapy and community-based aged care assessment. The latter is carried out by the ACAT [22]. At the time the study was conducted, the Fremantle ACAT was incorporated within and administered by the DCGM. The ACAT is staffed by a full complement of doctors, nurses and allied health staff. Referrals to the DCGM are usually received from primary care doctors, but the ACAT also receives and acts on referrals from individuals and their families.

Four trained raters, comprising two clinical nurses, a social worker and an occupational therapist, assessed patients consecutively between July 2013 and August 2015. All raters were trained in the use of the instruments using the training modules developed at University College Cork. Examination took place at the patient’s home. The raters assessed the patient according to our standard comprehensive process and then rated the CARI instrument. For each patient, 40 items (elements covering different domains of the CARI assessment) were collected [23]. All items are measured using collapsing domains. If there is a concern regarding a domain, then each of the items is scored. Where no concern exists, the item receives a score of zero and the next item is scored. Where there is concern, the issue it relates to is scored from 1 (mild) to 3 (severe), representing increasing concern. Descriptors of each level of concern are provided to assist raters. The CARI covers seven mental state items, fifteen ADLs items and nine medical state items (see Supplementary Materials). Once the domains and their respective items, where required, are scored, the *Caregiver Network Score* for each of the three domains, taking account of items rated, is scored for all three domains [27]. Ratings for this are measured on a 5-point Likert scale with scores 1 (can manage) to 5 (absent/liability). A score of 1 indicates that the patient’s caregiver network manages the care needs well [27]. Higher scores reflect increasing inability of the caregiver network to cope with the patient’s care needs.

Frailty was scored using the Clinical Frailty Scale (CFS), a widely-used, global, subjective, pictorial, and clinical measure of frailty in older adults based on their functional level and ability to manage ADL, incorporating cognition scored from 1 (very fit) to 9 (terminally ill) [28], which is widely validated in community-dwelling older adults [29].

### 2.3. Ethics and Statistical Analysis

The South Metropolitan Health Service, Human Research Ethics Committee, approved this study as a quality assurance project (reference ac. 13/6). The ethics committee did not require study-specific consent as this work was conducted as part of an ongoing quality improvement project. During the assessment period, 636 patients were assessed, and of these, 417 had outcome data available and were included. Twelve months after the initial assessment, the hospital database was checked to determine if the patient was still alive. A record of hospital admissions in the public system was readily accessible. We contacted patients’ primary doctor for information about private hospital admissions. The patient or their carer was then contacted to obtain additional information including whether patients had been institutionalised.

All data were examined using SPSS V20. As most data were non-normally distributed, continuous variables were analysed using the Mann–Whitney test. Categorical variables were compared with the chi-square test. Correlations were derived using Spearman coefficients. To facilitate analysis RISC scores, *Global Risk Scores* were categorised into low (scores of 1 or 2), medium (score of 3) and high (scores of 4 or 5). Accuracy for the three adverse outcomes at one year for the RISC and CFS were assessed from the area under the curve (AUC) of receiver operating characteristic curves with their 95% confidence intervals (CI). AUC values between 0.60 and 0.69 are regarded as poor, between 0.70 and 0.79 as fair/modest, between 0.80 and 0.89 as good and ≥0.90 as excellent. Binary logistic regression was used to examine the strength of association between independent variables and healthcare outcomes.

## 3. Results

Examining the 417 patients included in this analysis, the sample consisted of 257 females (61.6%) and 160 males (38.4%). Their mean age was 81.50 years, standard deviation (sd) ±7.37, range 54–101 and a median age of 82 (interquartile 77–87) years. Baseline characteristics are presented in Table 1. The distribution of RISC scores in the sample is also presented in Table 1. At the 12-month follow-up, 94 (22.5%) patients had been institutionalised in long-term (residential) care (LTC) facilities, 186 (44.6%) had been admitted at least overnight to hospital and 41 (9.8%) had died. Analysis of patient outcomes showed that older age was statistically significantly associated with institutionalisation (*p* = 0.002) but not with hospitalisation (*p* = 0.06) or death (*p* = 0.12). This was predominantly a frail sample with a median CSF score of 6 and interquartile range (IQR) of 5–6. The CFS score was significantly associated with all three adverse outcomes: institutionalisation (*p* = 0.001), hospitalisation (*p* = 0.007) and death (*p* < 0.001).

The RISC scores were also statistically significantly associated with all three adverse outcomes at 12 months (*p* < 0.001), see Table 2. Of those institutionalised, 46.8% had a high RISC score, and 7.4% had a low RISC score. Of those hospitalised, 77.4% were assessed as either medium or high RISC scores. For risk of death, 78.1% were assessed as medium or high RISC scores. Analysis of the domain scores showed that ADLs predicted institutionalisation (*p* < 0.001) and death (*p* = 0.01), while the mental state domain predicted institutionalisation (*p* < 0.001) and the medical state predicted hospitalisation (*p* < 0.001).

Correlations between the *Global Risk Scores* and the three adverse outcomes are listed in Table 3. The ADL domain had the strongest correlation with institutionalisation, r = 0.51 (95% CI: 0.43–0.58), while the medical state domain had moderate correlation with hospitalisation, 0.44 (0.35–0.51). The CFS score also had moderate correlation with hospitalisation r = 0.44 (0.35–0.52) and death 0.51 (0.43–0.58).

Table 4 provides AUC values for the CFS and RISC with its domains for each outcome, showing that the *Global Risk Score* for institutionalisation indeed best predicted institutionalisation with an AUC of 0.76 (95% CI: 0.71–0.81), while the *Global Risk Score* for death risk was most accurate for mortality, 0.74 (0.66–0.83). Neither the CFS nor RISC scores were accurate in predicting hospitalisation, with the highest AUC for the *Global Risk Score* for hospitalisation of AUC 0.62 (0.56–0.67).

Adjusting for age and sex, frailty remained independently associated with one-year mortality, odds ratio (OR) 2.68, 95% confidence interval (CI): 1.77–4.06 (*p* < 0.001). It was also independently associated with hospitalisation (OR 1.4, 95% CI: 1.09–1.82, *p* = 0.008) and institutionalisation (OR 1.51, 95% CI: 1.11–2.05, *p* = 0.01). Similarly, the RISC was independently associated with death (OR 3.1, 95% CI: 2.12–4.53, *p* < 0.001), hospital admission (OR 1.73, 95% CI: 1.34–2.25, *p* < 0.001) and LTC (OR 3.48, 95% CI: 2.52–4.83, *p* < 0.001).

## 4. Discussion

This paper presents the frequency and distribution of several established predictors of functional decline and frailty among community-dwelling older adults being assessed by a multidisciplinary team led by specialist nurses in the community (ACAT) as part of the CARTS programme in Western Australia. It also presents the strength of the association between frailty and factors that contribute to the perceived risk of and then actual frequency of three common adverse outcomes at one year (institutionalisation, hospitalisation and death), scored using recently developed, validated screening and assessment instruments, the RISC and CARI. The results suggest that there were high levels of complexity and high levels of both perceived risk of the three important healthcare outcomes and of actual event rates, mirroring the high levels of frailty (mean CFS score 5.74) among this cohort of older community dwellers. While higher than frailty prevalence proportions reported in other studies [2], this was expected given that patients who are assessed by the ACAT are a selected sample of older adults and are more likely to have medical and other comorbidities than a cross-sectional sample of all community-dwelling older adults [30,31,32]. Indeed, referrals to the ACAT are for older adults with frailty and disability for whom the referrer believes that care needs are unmet.

In this cohort of referred older adults, the risk of adverse outcomes as assessed by experienced clinicians was high as determined by the combined medium- and high-risk designations on the RISC. Perceived risk of hospitalisation was higher than for the other adverse outcomes. This is unsurprising given that it is a higher frequency outcome than death and institutionalisation. In this sample, almost one-third (29%) of patients were recorded as having severe concerns over their ability to manage ADLs, with a much smaller proportion having severe concerns for their mental state including cognition (12.9%) or medical comorbidities (4.6%). Those institutionalised had statically significantly higher scores, indicating greater concerns for ADLs and mental state issues but not for medical issues. Those who were hospitalised were significantly more likely to have concerns over their medical but not ADL or mental states. Only ADL scores were significantly higher among those who died at one year, in keeping with evidence that functional limitations are associated with mortality [33], even in those aged 50–64 years [34]. Here, ADL impairments correlated most with institutionalisation and death, while medical comorbidities correlated with hospitalisation. Frailty measured using the CFS also had moderate correlation with institutionalisation, hospitalisation and death, as has been shown in a recent systematic review [35]. In this study, after adjusting for age and sex, frailty was associated with 2.5 times the odds of one-year mortality and approximately 1.5 greater odds of being admitted to hospital or LTC within the next year.

The results show that RISC scores and the CFS were significantly associated with most adverse outcomes and had similar accuracy, which is comparable to previously published research comparing both scales in Ireland [10]. The *Global Risk Score* for institutionalisation was, however, in this sample significantly more accurate than the CFS in predicting those that were admitted to long-term care at one year. The results also reaffirm the challenges of predicting those who will be hospitalised, which in several studies is difficult to predict with AUC values usually around or <0.70 [4,36,37,38] even where new machine learning algorithms are applied [39,40]. It is suggested that predictor effect heterogeneity and differing baseline risk may partly explain the limited accuracy of existing prediction models [41]. There are also significant differences in whether physicians will decide to admit patients to hospital, though while clinician risk tolerance may also be a factor, there is little evidence to support this [42]. In this study, the RISC score had good accuracy for one-year mortality (AUC of 0.74), which is similar to that found in other studies using the RISC [10]. It was markedly better than the CFS, which had low predictive validity in this study (AUC 0.69). This is similar to other studies examining the accuracy of the CFS for mortality at one year, e.g., amongst older adults attending the emergency department [43], though relatively few studies before this and the studies comparing the CFS to the RISC have examined the use of the CFS scored in the patients’ home environment. The evidence for the CFS amongst hospitalised inpatients suggests it may be more accurate in this setting [44,45]. The RISC is also comparable or better than a variety of measures including the Timed Up and Go Test, cognitive screens and measures of frailty (Frailty Index or Physical Frailty) in longitudinal studies of community-dwelling older adults [46].

This paper has several limitations. Follow-up data were not available for 219 patients, so the results may not be representative of the entire cohort. As this was designed as a research study within a quality improvement project, a sample size calculation was not performed a priori. Hence, it is possible this study is underpowered to show the true diagnostic performance of the screening instruments compared. There is also risk of selection bias given that only disabled older adults in the community proceed to an ACAT assessment. Hence, while representing real-life practice in this setting, it is possible that the results are not representative of all older adults assessed in the community by nurses and other members of the interdisciplinary team. Additionally, it is not certain that all assessors correctly classified patients according to their risk. Although training was provided to raters, this is the first validation of the RISC and CFS in community settings in Australia, and neither has been validated with aged care clinicians in Australia, which may have led to bias. Although we assessed the inter-rater reliability of the RISC, the reliability of the CFS was not determined. The subjectivity of the frailty assessment is another limitation. Inter-rater reliability was not assessed formally, though standardised training was provided for raters who already had extensive experience measuring frailty and conducting CGA. Both the assessor’s own assessment of frailty and the CFS are subjective measures, and the inclusion of an objective observer-rated assessment instrument would have reduced potential bias. This said, the CFS is a widely used instrument with excellent reliability and validity for frailty [28,29]. Further, frailty as determined by the CFS was based on CGA, recognised as a gold standard for measuring frailty in clinical practice and research studies [47,48]. Another limitation is that frailty was applied to some middle-aged patients including those aged ≤60 years of age. However, applying frailty models to younger patients, while controversial with limited evidence [49], is accepted by some researchers [50], and while it shares many risk factors with late-life frailty, it appears to also be particularly associated with mental health conditions and pain syndromes [51]. While scored as frail in our study, these patients may not necessarily have scored positive for frailty if other competing models of frailty, such as the frailty phenotype or the multidimensional deficit accumulation approach, were used instead [52]. This said, frailty is a heterogenous concept and the CFS is widely used as a global measure, which correlates with and can incorporate both physical and deficit accumulation models [28,53]. An alternative is that what was captured as frailty may instead have represented sarcopenia, a different syndrome closer to physical frailty, which was not considered in this study. This often co-occurs with frailty and can be difficult to distinguish [54]. Future studies and clinicians working in ACATs should consider including a validated measure of sarcopenia such as the SARC-F [55]. The strengths of this study are the comprehensive nature of the assessment and the accompanying medical record review, and the inclusion of a large cross-sectional and representative community sample, increasing the generalisability of the results.

The RISC was developed to measure patients’ risk levels, and this study provides further external validation of the instrument, highlighting that it is accurate in predicting the likelihood of future adverse outcomes in a community-dwelling older sample of Australians. Future studies will investigate if the RISC, aligned to tailored intervention programmes or care packages as part of the CARTS programme, can reduce risk and the incidence of adverse outcomes in community-dwelling older adults. It would also be important to compare the efficacy of the service model examined in this paper with other service models both in Australia and in diverse service systems or cultures. As part of this and any future study, surveys and interviews with both healthcare staff and end users would help to refine the approach and model. Finally, it should also be compared to other measures of frailty and emerging machine learning- and artificial intelligence-powered algorithms [56,57,58].

## 5. Conclusions

In this study, a significant proportion of referred community-dwelling older adults were perceived by an experienced clinician to be at medium or high risk of adverse outcomes. Follow-up 12 months later showed that the initial assessment correlated well with outcomes. Previous work has shown that factors traditionally associated with frailty such as age, gender and social isolation do not correlate with the CFS, RISC or clinician perception of risk of adverse outcomes, suggesting that despite their high prevalence, they may not be useful indicators for risk assessment. Further research in other aged care settings is needed to determine if the RISC should be used more widely.

## Figures and Tables

**Table 1:** Descriptive characteristics of the study sample (n = 417).

Demographic Characteristics	Mean ± SD/Median (IQR) Range n (%)
Age (years)	Mean 81.50 ± 7.37/median 82 (77–87) Range 54–101
Biological sex	
- Males (%)	160 (38.4%)
- Females (%)	257 (61.6%)
Baseline assessment characteristics	
Clinical Frailty Scale score	Mean 5.74 ± 0.83/median 6 (5–6) Range 2–9
*RISC Scores*	
*Mental State Concerns*	
- No concern	43 (10.3%)
- Mild concern	142 (34.1%)
- Moderate concern	178 (42.7%)
- Severe concern	54 (12.9%)
*ADLs*	
- No concern	4 (1.0%)
- Mild concern	52 (12.5%)
- Moderate concern	240 (57.6%)
- Severe concern	121 (29.0%)
*Medical State Concerns*	
- No concern	99 (23.9%)
- Mild concern	220 (53.0%)
- Moderate concern	77 (18.6%)
- Severe concern	19 (4.6%)
*Domain Sub-scores*	
Mental state (based on 7 components)	Mean 5.39 ± 3.87/median 5 (2–8) Range 0–19
ADLs (based on 15 components)	Mean 18.07 ± 7.76/median 18 (13–23) Range 1–44
Medical state (based on 9 components)	Mean 5.89 ± 2.26/median 6 (4–7) Range 0–14
*Global Risk Scores* Institutionalisation Global Risk Scores	
- Low (score 1–2)	159 (38.1%)
- Medium (score 3)	170 (40.8%)
- High (score 4–5)	88 (21.1%)
*Hospitalisation Global Risk Scores*	
- Low (score 1–2)	126 (30.2%)
- Medium (score 3)	211 (50.6%)
- High (score 4–5)	80 (19.2%)
*Death Global Risk Scores*	
- Low (score 1–2)	233 (55.9%)
- Medium (score 3)	149 (35.7%)
- High (score 4–5)	35 (8.4%)
Actual (1-year) outcomes	
Institutionalised	94 (22.5%)
Hospitalised	186 (44.6%)
Dead	41 (9.8%)

ADL = activities of daily living; IQR = interquartile range; RISC = Risk Instrument for Screening in the Community; SD = standard deviation.

**Table 2:** Characteristics of patients (n = 417) according to each Risk Instrument for Screening in the Community (RISC) domain, *Global Risk Scores* (institutionalisation, hospitalisation and death) and by actual outcome at one year.

Characteristic (Median ± IQR) or %	Institutionalised	Not Institutionalised	*p*-Value	Hospitalised	Not Hospitalised	*p*-Value	Dead	Alive	*p*-Value
Age	84 ± 8	82 ± 10	0.002	84 ± 11	81 ± 9	0.06	85 ± 12	82 ± 10	0.12
Female (%)	68.1%	59.8%	0.14	59.7%	63.2%	0.46	56.1%	62.2%	0.44
Clinical Frailty Scale	6 ± 0	6 ± 1	0.001	6 ± 1	6 ± 1	0.007	6 ± 1	6 ± 1	<0.001
RISC Domain									
ADLs *	20 ± 10	17 ± 11	<0.001	19 ± 11	17 ± 10	0.10	22 ± 13	18 ± 10	0.01
Mental state **	7 ± 7	4 ± 6	<0.001	4 ± 6	5 ± 6	0.27	3 ± 6	5 ± 6	0.11
Medical state ***	6 ± 2	6 ± 3	0.18	6 ± 3	5 ± 3	<0.001	6 ± 3	6 ± 3	0.26
*Global Risk Score* for Institutionalisation									
Low, n = 159 (%)	7.4%	47.1%		34.9%	40.7%		22.0%	39.9%	
Medium, n = 170 (%)	45.7%	39.3%		45.2%	37.2%		41.5%	40.7%	
High, n = 88 (%)	46.8%	13.6%	<0.001	19.9%	22.1%	0.26	36.5%	19.4%	0.02
*Global Risk Score* for Hospitalisation									
Low, n = 126 (%)	21.3%	32.8%		22.6%	36.4%		7.4%	32.7%	
Medium, n = 211 (%)	57.4%	48.6%		50.5%	50.6%		46.3%	51.1%	
High, n = 80 (%)	21.3%	18.6%	0.10	26.9%	13.0%	<0.001	46.3%	16.2%	<0.001
*Global Risk Score* for Death									
Low, n = 233 (%)	52.1%	57.0%		48.9%	61.5%		22.0%	59.6%	
Medium, n = 149 (%)	39.4%	34.7%		38.7%	33.3%		41.5%	35.1%	
High, n = 35 (%)	8.5%	8.4%	0.69	12.4%	5.2%	0.01	36.6%	5.3%	<0.001

ADL = activities of daily living; * based on 15 components, ** based on 7 components, *** based on 9 components.

**Table 3:** Correlations with 95% confidence intervals between Global Risk Instrument for Screening in the Community (RISC) scores and the Clinical Frailty Scale (CFS) and other components of the RISC (Severity and Domain sub-scores).

Assessment	*Global Risk Score* for Institutionalisation	*Global Risk Score* for Hospitalisation	*Global Risk Score* for Death
**CFS score (range 1–9)**	0.42 * [0.33 to 0.49]	0.44 * [0.35 to 0.52]	0.51 * [0.43 to 0.58]

**RISC Severity of Concern**			
Mental state	0.19 * [0.09 to 0.28]	−0.04 [−0.14 to 0.06]	−0.03 [−0.13 to 0.07]
ADLs	0.37 * [0.28 to 0.45]	0.16 * [0.06 to 0.25]	0.17 * [0.08 to 0.27]
Medical state	0.27 * [0.16 to 0.37]	0.18 * [0.07 to 0.29]	0.27 * [0.16 to 0.37]
**RISC Domain Subscores**			
ADLs (15 components)	0.51 * [0.43 to 0.58]	0.34 * [0.25 to 0.42]	0.42 * [0.34 to 0.50]
Mental state (7 components)	0.29 * [0.19 to 0.38]	−0.09 [−0.18 to 0.01]	−0.05 [−0.15 to 0.04]
Medical state (9 components)	0.31 * [0.22 to 0.39]	0.44 * [0.35 to 0.51]	0.41 * [0.32 to 0.49]

* Indicates that correlation coefficient is statistically significant at 0.05 level.

**Table 4:** Area under the curve (AUC) with 95% confidence intervals (CI) for predicting actual outcomes at one year comparing the Clinical Frailty Scale and Risk Instrument for Screening in the Community (RISC).

Measure	Predicting Actual Outcome		
	Institutionalisation (AUC and 95% CI)	Hospitalisation (AUC and 95% CI)	Death (AUC and 95% CI)
**Clinical Frailty Scale score**	0.60 (0.54–0.66)	0.58 (0.52–0.63)	0.69 (0.61–0.77)
**RISC Severity sub-score**			
Mental state	0.62 (0.55–0.68)	0.48 (0.42–0.53)	0.42 (0.33–0.52)
ADLs	0.58 (0.52–0.65)	0.53 (0.47–0.58)	0.51 (0.41–0.60)
Medical state	0.60 (0.53–0.67)	0.51 (0.45–0.57)	0.52 (0.43–0.62)
**RISC Domain sub-score**			
ADLs (based on 15 components)	0.64 (0.58–0.70)	0.55 (0.49–0.61)	0.61 (0.52–0.71)
Mental state (based on 7 components)	0.65 (0.58–0.71)	0.47 (0.41–0.53)	0.43 (0.33–0.52)
Medical state (based on 9 components)	0.54 (0.47–0.60)	0.62 (0.56–0.67)	0.59 (0.50–0.68)
***Global Risk Score***			
Institutionalisation	0.76 (0.71–0.81)	0.53 (0.47–0.59)	0.62 (0.54–0.71)
Hospitalisation	0.56 (0.49–0.62)	0.61 (0.56–0.67)	0.71 (0.62–0.79)
Death	0.52 (0.46–0.59)	0.58 (0.52–0.64)	0.74 (0.66–0.83)

ADLs = activities of daily living.

## Data Availability

Data are available on request.

## Supplementary Materials

The following supporting information can be downloaded at: https://www.mdpi.com/article/10.3390/healthcare12131339/s1, Figure S1: The Risk Instrument Screening in the Community (RISC); copyrighted and available at: http://www.biomedcentral.com/1471-2318/14/104/figure/F1 (accessed on 24 June 2024); Figure S2: The Community Assessment of Risk Instrument (CARI). Copyrighted and available at: http://www.researchgate.net/publication/308745424 (accessed on 24 June 2024).

## References

[1] Metzelthin S.F., Daniels R., van Rossum E., de Witte L., van den Heuvel W.J.A., Kempen G.I.J.M. (2010). The psychometric properties of three self-report screening instruments for identifying frail older people in the community. BMC Public Health.

[2] O’Caoimh R., Sezgin D., O’Donovan M.R., Molloy D.W., Clegg A., Rockwood K., Liew A. (2021). Prevalence of frailty in 62 countries across the world: A systematic review and meta-analysis of population-level studies. Age Ageing.

[3] Kojima G., Iliffe S., Walters K. (2018). Frailty index as a predictor of mortality: A systematic review and meta-analysis. Age Ageing.

[4] Kansagara D., Englander H., Salanitro A., Kagen D., Theobald C., Freeman M., Kripalani S. (2011). Risk prediction models for hospital readmission: A systematic review. JAMA.

[5] O’Caoimh R., Cornally N., Weathers E., O’Sullivan R., Fitzgerald C., Orfila F., Clarnette R., Paúl C., Molloy D.W. (2015). Risk prediction in the community: A systematic review of case-finding instruments that predict adverse healthcare outcomes in community-dwelling older adults. Maturitas.

[6] Veerman J., Barendregt J., Mackenbach J. (2005). Quantitative health impact assessment: Current practice and future directions. J. Epidemiol. Community Health.

[7] Alba A.C., Agoritsas T., Walsh M., Hanna S., Iorio A., Devereaux P.J., McGinn T., Guyatt G. (2017). Discrimination and Calibration of Clinical Prediction Models: Users’ Guides to the Medical Literature. JAMA.

[8] Vermeulen J., Neyens J.C., van Rossum E., Spreeuwenberg M.D., de Witte L.P. (2011). Predicting ADL disability in community-dwelling elderly people using physical frailty indicators: A systematic review. BMC Geriatr..

[9] Carpenter C.R., Shelton E., Fowler S., Suffoletto B., Platts-Mills T.F., Rothman R.E., Hogan T.M. (2015). Risk factors and screening instruments to predict adverse outcomes for undifferentiated older emergency department patients: A systematic review and meta-analysis. Acad. Emerg. Med..

[10] O’Caoimh R., Gao Y., Svendrovski A., Healy E., O’Connell E., O’Keeffe G., Cronin U., Igras E., O’Herlihy E., Fitzgerald C. (2015). The Risk Instrument for Screening in the Community (RISC): A new instrument for predicting risk of adverse outcomes in community dwelling older adults. BMC Geriatr..

[11] Shah S.J., Fang M.C., Wannier S.R., Steinman M.A., Covinsky K.E. (2022). Association of Social Support with Functional Outcomes in Older Adults Who Live Alone. JAMA Intern. Med..

[12] de Vriesm N.M., Staal J.B., van Ravensberg C.D., Hobbelen J.S.M., Olde Rikkerte M.G.M., van der Nijhuis-Sanden M.W.G. (2011). Outcome instruments to measure frailty: A systematic review. Ageing Res. Rev..

[13] Clegg A., Young J., Iliffe S., Rikkert M.O., Rockwood K. (2013). Frailty in elderly people. Lancet.

[14] Salvi F., Morichi V., Grilli A., Lancioni L., Spazzafumo L., Polonara S., Abbatecola A.M., De Tommaso G., Dessi-Fulgheri P., Lattanzio F. (2012). Screening for frailty in elderly emergency department patients by using the Identification of Seniors at Risk (ISAR). J. Nutr. Health Aging.

[15] Roberts H.C., Hemsley Z.M., Thomas G., Meakins P., Powell J., Robison J., Gove I., Turner G., Sayer A.A. (2006). Nurse-led implementation of the single assessment process in primary care: A descriptive feasibility study. Age Ageing.

[16] Jansen-Kosterink S., van Velsen L., Frazer S., Dekker-van Weering M., O’Caoimh R., Vollenbroek-Hutten M. (2019). Identification of community-dwelling older adults at risk of frailty using the PERSSILAA screening pathway: A methodological guide and results of a large-scale deployment in the Netherlands. BMC Public Health.

[17] Stoop A., Lette M., van Gils P.F., Nijpels G., Baan C.A., de Bruin S.R. (2019). Comprehensive geriatric assessments in integrated care programs for older people living at home: A scoping review. Health Soc. Care Community.

[18] Coburn K.D., Marcantonio S., Lazansky R., Keller M., Davis N. (2012). Effect of a community-based nursing intervention on mortality in chronically Ill older adults: A randomized controlled trial. PLoS Med..

[19] Trapp-Moen B., Tyrey M., Cook G., Heyman A., Fillenbaum G.G. (2001). In-home assessment of dementia by nurses: Experience using the CERAD evaluations. Gerontologist.

[20] Ballard J., Mooney M., Dempsey O. (2013). Prevalence of frailty-related risk factors in older adults seen by community nurses. J. Adv. Nurs..

[21] McDonald A., Frazer K., Warters A. (2022). Irish Public Health Nursing Services and Home Support Services: Governance of older persons’ home care. Public Health Nurs..

[22] Clarnette R., Goh M., Bharadwaj S., Ryan J., Ellis S., Svendrovski A., Molloy D.W., O’Caoimh R. (2019). Screening for cognitive impairment in an Australian aged care assessment team as part of comprehensive geriatric assessment. Neuropsychol. Dev. Cogn. B Aging Neuropsychol. Cogn..

[23] Clarnette R.M., Ryan J.P., O’Herlihy E., Svendrovski A., Cornally N., O’Caoimh R., Leahy-Warren P., Paul C., Molloy D.W. (2015). The Community Assessment of Risk Instrument: Investigation of Inter-Rater Reliability of an Instrument Measuring Risk of Adverse Outcomes. J. Frailty Aging.

[24] O’ Caoimh R., Weathers E., Hally R., O’ Sullivan R., Fitzgerald C., Cornally N., Svendrovski A., Healy E., O’Connell E., O’keeffe G., Helfert M., Holzinger A., Ziefle M., Fred A., O’Donoghue J., Röcker C. (2015). The community assessment of risk and treatment strategies (CARTS): An integrated care pathway to manage frailty and functional decline in community dwelling older adults. Communications in Computer and Information Science.

[25] Weathers E., O’Caoimh R., O’Sullivan R., Paúl C., Orfilia F., Clarnette R., Fitzgerald C., Svendrovski A., Cornally N., Leahy-Warren P. (2016). The inter-rater reliability of the Risk Instrument for Screening in the Community. Br. J. Community Nurs..

[26] Santos S., O’Caoimh R., Teixeira L., Alves S., Molloy W., Paúl C. (2021). Validation of the Portuguese Version of the Risk Instrument for Screening in the Community (RISC) Among Older Patients in Primary Care in Northern Portugal. Front. Public Health.

[27] O’Caoimh R., Cornally N., Svendrovski A., Weathers E., FitzGerald C., Healy E., O’Connell E., O’Keeffe G., O’Herlihy E., Gao Y. (2015). Measuring the Effect of Carers on Patients’ Risk of Adverse Healthcare Outcomes Using the Caregiver Network Score. J. Frailty Aging.

[28] Rockwood K., Song X., MacKnight C., Bergman H., Hogan D.B., McDowell I., Mitnitski A. (2005). A global clinical measure of fitness and frailty in elderly people. CMAJ.

[29] Church S., Rogers E., Rockwood K., Theou O. (2020). A scoping review of the Clinical Frailty Scale. BMC Geriatr..

[30] Collard R.M., Boter H., Schoevers R.A., Oude Voshaar R.C. (2012). Prevalence of frailty in community-dwelling older persons: A systematic review. J. Am. Geriatr. Soc..

[31] To T.L., Doan T.N., Ho W.C., Liao W.C. (2022). Prevalence of Frailty among Community-Dwelling Older Adults in Asian Countries: A Systematic Review and Meta-Analysis. Healthcare.

[32] Siriwardhana D.D., Hardoon S., Rait G., Weerasinghe M.C., Walters K.R. (2018). Prevalence of frailty and prefrailty among community-dwelling older adults in low-income and middle-income countries: A systematic review and meta-analysis. BMJ Open.

[33] Gao Y., Du L., Cai J., Hu T. (2023). Effects of functional limitations and activities of daily living on the mortality of the older people: A cohort study in China. Front. Public Health.

[34] Brown R.T., Diaz-Ramirez L.G., Boscardin W.J., Lee S.J., Williams B.A., Steinman M.A. (2019). Association of Functional Impairment in Middle Age With Hospitalization, Nursing Home Admission, and Death. JAMA Intern. Med..

[35] Falk Erhag H., Guðnadóttir G., Alfredsson J., Cederholm T., Ekerstad N., Religa D., Nellgård B., Wilhelmson K. (2023). The Association Between the Clinical Frailty Scale and Adverse Health Outcomes in Older Adults in Acute Clinical Settings—A Systematic Review of the Literature. Clin. Interv. Aging.

[36] Donzé J., Aujesky D., Williams D., Schnipper J.L. (2013). Potentially avoidable 30-day hospital readmissions in medical patients: Derivation and validation of a prediction model. JAMA Intern. Med..

[37] Klunder J.H., Panneman S.L., Wallace E., de Vries R., Joling K.J., Maarsingh O.R., van Hout H.P.J. (2022). Prediction models for the prediction of unplanned hospital admissions in community-dwelling older adults: A systematic review. PLoS ONE.

[38] Marcusson J., Nord M., Dong H.J., Lyth J. (2020). Clinically useful prediction of hospital admissions in an older population. BMC Geriatr..

[39] Morgan D.J., Bame B., Zimand P., Dooley P., Thom K.A., Harris A.D., Bentzen S., Ettinger W., Garrett-Ray S.D., Tracy J.K. (2019). Assessment of Machine Learning vs Standard Prediction Rules for Predicting Hospital Readmissions. JAMA Netw. Open.

[40] Feretzakis G., Karlis G., Loupelis E., Kalles D., Chatzikyriakou R., Trakas N., Karakou E., Sakagianni A., Tzelves L., Petropoulou S. (2022). Using Machine Learning Techniques to Predict Hospital Admission at the Emergency Department. J. Crit. Care Med..

[41] Meid A.D., Gonzalez-Gonzalez A.I., Dinh T.S., Blom J., van den Akker M., Elders P., Thiem U., Küllenberg de Gaudry D., Swart K.M.A., Rudolf H. (2021). Predicting hospital admissions from individual patient data (IPD): An applied example to explore key elements driving external validity. BMJ Open.

[42] Smulowitz P.B., Burke R.C., Ostrovsky D., Novack V., Isbell L., Kan V., Landon B.E. (2024). Clinician Risk Tolerance and Rates of Admission from the Emergency Department. JAMA Netw. Open.

[43] Rueegg M., Nissen S.K., Brabrand M., Kaeppeli T., Dreher T., Carpenter C.R., Bingisser R., Nickel C.H. (2022). The clinical frailty scale predicts 1-year mortality in emergency department patients aged 65 years and older. Acad. Emerg. Med..

[44] Soh C.H., Lim W.K., Reijnierse E.M., Maier A.B. (2023). Clinical frailty scale score during geriatric rehabilitation predicts short-term mortality: RESORT cohort study. Ann. Phys. Rehabil. Med..

[45] Bielza R., Balaguer C., Zambrana F., Arias E., Thuissard I.J., Lung A., Oñoro C., Pérez P., Andreu-Vázquez C., Neira M. (2022). Accuracy, feasibility and predictive ability of different frailty instruments in an acute geriatric setting. Eur. Geriatr. Med..

[46] Cheong C.Y., Yap P., Gwee X., Chua D.Q.L., Wee S.L., Yap K.B., Ng T.P. (2021). Physical and functional measures predicting long-term mortality in community-dwelling older adults: A comparative evaluation in the Singapore Longitudinal Ageing Study. Aging.

[47] Lee H., Lee E., Jang I.Y. (2020). Frailty and Comprehensive Geriatric Assessment. J. Korean Med. Sci..

[48] Moloney E., O’Donovan M.R., Sezgin D., Flanagan E., McGrath K., Timmons S., O’Caoimh R. (2023). Diagnostic Accuracy of Frailty Screening Instruments Validated for Use among Older Adults Attending Emergency Departments: A Systematic Review and Meta-Analysis. Int. J. Environ. Res. Public Health.

[49] Spiers G.F., Kunonga T.P., Hall A., Beyer F., Boulton E., Parker S., Bower P., Craig D., Todd C., Hanratty B. (2021). Measuring frailty in younger populations: A rapid review of evidence. BMJ Open.

[50] Gordon E.H., Peel N.M., Hubbard R.E., Reid N. (2023). Frailty in younger adults in hospital. QJM Int. J. Med..

[51] Bai G., Wang Y., Mak J.K.L., Ericsson M., Hägg S., Jylhävä J. (2023). Is Frailty Different in Younger Adults Compared to Old? Prevalence, Characteristics, and Risk Factors of Early-Life and Late-Life Frailty in Samples from Sweden and UK. Gerontology.

[52] Cesari M., Gambassi G., van Kan G.A., Vellas B. (2014). The frailty phenotype and the frailty index: Different instruments for different purposes. Age Ageing.

[53] Gordon S., Grimmer K., Baker N. (2020). Do two measures of frailty identify the same people? An age-gender comparison. J. Eval. Clin. Pract..

[54] Ligthart-Melis G.C., Luiking Y.C., Kakourou A., Cederholm T., Maier A.B., de van der Schueren M.A.E. (2020). Frailty, Sarcopenia, and Malnutrition Frequently (Co-)occur in Hospitalized Older Adults: A Systematic Review and Meta-analysis. J. Am. Med. Dir. Assoc..

[55] Malmstrom T.K., Miller D.K., Simonsick E.M., Ferrucci L., Morley J.E. (2016). SARC-F: A symptom score to predict persons with sarcopenia at risk for poor functional outcomes. J. Cachexia Sarcopenia Muscle.

[56] Olender R.T., Roy S., Nishtala P.S. (2023). Application of machine learning approaches in predicting clinical outcomes in older adults—A systematic review and meta-analysis. BMC Geriatr..

[57] Velazquez-Diaz D., Arco J.E., Ortiz A., Pérez-Cabezas V., Lucena-Anton D., Moral-Munoz J.A., Galán-Mercant A. (2023). Use of Artificial Intelligence in the Identification and Diagnosis of Frailty Syndrome in Older Adults: Scoping Review. J. Med. Internet Res..

[58] Sajeev S., Champion S., Maeder A., Gordon S. (2022). Machine learning models for identifying pre-frailty in community dwelling older adults. BMC Geriatr..

