# Supplementary material for: Predicting Outcomes in Frail Older Community-Dwellers in Western Australia: Results from the Community Assessment of Risk Screening and Treatment Strategies (CARTS) Programme
Source: Healthcare (Basel). 2024 Jul 5;12(13):1339. doi: 10.3390/healthcare12131339 (PMC11241488; doi:10.3390/healthcare12131339)
Supplement: Supplementary file 1 [file healthcare-12-01339-s001.zip › healthcare-3059607-supplementary.pdf]

Supplementary Material

Figure S1. The Risk Instrument Screening in the Community (RISC): Copyrighted and available at: The RISC is available at http://www.biomedcentral.com/1471-2318/14/104/figure/F1

RISC Score Sheet©

Demographics

Personal Details: Name____

Address ____
____
____

Gender : M ☐ F ☐ **DOB** / / **ID** ____________________

Living Arrangements:

Alone ☐ Spouse ☐
Child ☐
Other ____________

Instructions	Step 1	Step 2	Step 3
**Domain**	**Concern**		**Caregiver Network**
If NO concern for a Domain, move on to the next Domain. Complete all 4 domains	Is there concern about issues in this domain? (Circle **Y**es or **N**o) Then complete Step 2	Circle the present severity of the concern (Circle:1,2,3) **1. Mild. 2. Moderate. 3. Severe.** Then complete Step 3	Is the caregiver network able to manage (Circle:1,2,3,4 or 5) **1.Can manage 2.Carer strain 3.Some gaps 4.Cannot manage 5.Absent/liability**
**1. Mental State**	N Y ↓ →	1 2 3	1 2 3 4 5
**2. ADLs**	N Y ↓ →	1 2 3	1 2 3 4 5
**3. Medical/Physical State**	N Y ↓ →	1 2 3	1 2 3 4 5
**4. Other specify**________________	N Y ↓ →	1 2 3	1 2 3 4 5

Global Risk Score (circle 1,2,3,4 or 5)

A. **Institutionalisation** Overall risk of admission to long-term care {nursing home} in the next year.	**1** Minimal / rare	**2** Low / unlikely	**3** Moderate / possible	**4** High / likely	**5** Extreme / certain

B. **Hospitalisation** Risk of hospitalisation including prolonged admission or readmission in the next year.	**1** Minimal / rare	**2** Low / unlikely	**3** Moderate / possible	**4** High / likely	**5** Extreme / certain

C. **Death** Risk of death in the next year.	**1** Minimal / rare	**2** Low / unlikely	**3** Moderate / possible	**4** High / likely	**5** Extreme / certain

Global Risk Score Definitions

1. Minimal: Little or no serious consequence related to the risk / **Rare:** The event will almost never occur.
2. Low: Small impact from the risk, unlikely to cause serious harm / **Unlikely:** Low probability of the event occurring.
3. Moderate: Significant risk present / **Possible:** The event may occur but is infrequent or unlikely to occur soon.
4. High: Serious impact likely from the risk /**Likely:** High probability of the event occurring.
5. Extreme: Severe consequences likely /**Certain:** The event will almost certainly occur.

Figure S2. The Community Assessment of Risk Instrument (CARI). Copyrighted available at: http://www.jfrailtyaging.com/all-issues.html?article=231

The CARTS- Community Assessment of Risk Tool Strategies

CARI Score Sheets©

Demographics: Personal Details: Name____________________________Gender M□ F□ DOB / / MRN_____________
Reason for referral: ____Date of assessment________
Educational Level: Primary □ Secondary □ 3rd level □Other □____
Living Arrangements: Alone □Living with______________
Location: Own Home □ Others` home□ Sheltered Housing □Nursing home□ Other □_____________
Support: Informal: Yes □No□ hrs/day__days/week___ Family/partner□ Friend □ Neighbour □Other □______
Formal: Yes □No□ hrs/day__ days/week___PHN□ Day care□ Home help □ Meals on wheels □Respite□ Other□____
Carer burden : Primary carer_____________
Carer Burden Score- Mild (0-10) □ Mod (11-20)□ Severe(21-30)□
Medical History: Primary diagnosis____________________
Other diagnoses____
Healthcare use: No. A&E attendances (in the last year)____No of admissions (in the last year)____N/A □
Medication: Prescription meds___ Over the counter meds___
Frailty: (Your overall impression) Frail - Yes □ No□

Instructions	Step 1	Step 2	Step 3
**Domain**	**Concern**	**Status**	**Care Network**
**Mental State**	Is there concern about issues in this domain?(Circle Yes or No)	**Circle the present severity of the concern**<br>1. Mild.<br>2. Moderate.<br>3. Severe.	Can the caregiver network manage this concern for this domain?<br>1.Can manage<br>2.Carer strain<br>3.Some gaps<br>4.Cannot manage<br>5.Absent/liability
	N □ Y □<br>↓ →	If NO concern, move on to the next Domain, 2.<br>If YES complete each section A,B,C below	
**A. Thinking & Reasoning**	If NO concern, move on to next section until domain is complete		1 2 3 4 5<br>↓
Cognition	N □ Y □<br>↓ →	1 Mild cognitive impairment (memory loss without functional loss (typically SMMSE of >24).<br>2 Established early-mild dementia (typically SMMSE of 24-20).<br>3 Moderate to severe dementia, (typically SMMSE of < 20).	
Insight & Executive Function	N □ Y □<br>↓ →	1 Some loss of insight, difficulty planning<br>2 Greater loss of awareness, diminished capacity.<br>3 No insight or capacity (cognitive/functional), unaware of self/ health.	
**B. Behaviours**			
Agitation (restlessness)	N □ Y □<br>↓ →	1 Agitation has occurred in the past but not evident presently.<br>2 Agitation present but manageable / Infrequent<br>3 Agitation present, wandering/restless, difficult to manage.	
Aggression (Physical)	N □ Y □<br>↓ →	1 Aggression has occurred in the past but not evident presently<br>2 Aggression present, but can be managed/ isolated episode(s).<br>3 Aggression difficult to manage- frequent outbursts.	
Risky Behaviours including Self neglect	N □ Y □<br>↓ →	1 Behaviours have occurred in the past but not recently.<br>2 Behaviours noted recently but can be managed.<br>3 Behaviours ongoing / difficult to manage.	
**C. Psychiatric**			
Anxiety /Depression	N □ Y □<br>↓ →	1 Past history/Some mild anxiety-depression symptoms.<br>2 Symptoms causing distress /social withdrawal.<br>3 Symptoms interfering with function.	
Delusions /Hallucinations /paranoia	N □ Y □<br>↓ →	1 History of delusions/ hallucinations. None recently.<br>2 Evidence of delusions/hallucinations/ but no distress.<br>3 Symptoms causing distress and/or interfering with function.	
**D. Other**	N □ Y □	Specify ________________________________<br>1 2 3	

Domain 2.<br>Issues	Step 1<br>Concern	Step 2<br>Status	Step 3<br>Care Network
**ADLs**<br>Activities of daily living	Is there concern about issues in this domain?(Circle Yes or No)	**Circle the present level of function**<br>**1. Supervision or Set up.**<br>**2. Assist.**<br>**3. Dependent.**	Can the caregiver network manage the concern for this domain?<br>**1.Can manage**<br>**2.Carer strain**<br>**3.Some gaps**<br>**4.Cannot manage**<br>**5.Absent/liability**
	N ☐ Y ☐<br>↓ →	If NO concern, move on to next Domain, 3.<br>If YES complete each section A, B below.	
**A. Basic ADLs**	If NO concern, move on to next section until domain is complete.		
Bladder	N ☐ Y ☐<br>↓ →	**1** Occasional incontinence e.g. once per week /situational.<br>**2** Frequently incontinent / wears pads.<br>**3** Completely incontinent, needs physical help with pads or toilet.	
Bowel	N ☐ Y ☐<br>↓ →	**1** Occasional incontinence e.g. once per week /situational.<br>**2** Frequently incontinent / wears pads.<br>**3** Completely incontinent, needs physical help with pads or toilet.	
Transfer	N ☐ Y ☐<br>↓ →	**1** Minor help/ standby assistance of one person/requires raised toilet seat or handrails.<br>**2** Major help / assistance of one to two people.<br>**3** Hoist / bed bound.	
Mobility	N ☐ Y ☐<br>↓ →	**1** Uses aid (stick/frame) or standby assistance one person.<br>**2** Major help / assistance of one to two people.<br>**3** Immobile.	
Dressing	N ☐ Y ☐<br>↓ →	**1** Can dress with supervision or set up/ Rarely changes cloths.<br>**2** Can dress upper half (but not lower half).<br>**3** Full assistance (upper & lower half) or resistive or refusing.	
Bathing	N ☐ Y ☐<br>↓ →	**1** Supervision in shower /bath but wash themselves/ Not washing.<br>**2** Needs assistance with set up.<br>**3** Full assistance or unable as resistive or refusing.	
Stairs/steps<br>(No stairs/ not used ☐)	N ☐ Y ☐<br>↓ →	**1** Needs supervision on stairs but can use stairs/ requires handrails.<br>**2** Physical assistance of one to two people up & down.<br>**3** Unable/needs stair-lift/unwilling to move downstairs but unsafe.	
Feeding	N ☐ Y ☐<br>↓ →	**1** Supervision /encouragement eating /set up.<br>**2** Needs some assistance e.g. cutting up food but patient can feed themselves.<br>**3** Hand fed/ not eating or refusing food / peg feeding.	**1 2 3 4 5**<br>↓
**B.Instrumental**			
Technology use	N ☐ Y ☐<br>↓ →	**1** Difficulty learning how or cannot use new appliances<br>**2** Can use with assistance/passive user (e.g. can answer phone but cannot initiate).<br>**3** Unable / using inappropriately (calling at night).	
Shopping	N ☐ Y ☐<br>↓ →	**1** Needs someone to plan shopping with them / help with bags.<br>**2** Needs someone to plan/physically assist them with shopping.<br>**3** Unable to shop, would need shopping delivered.	
Food preparation	N ☐ Y ☐<br>↓ →	**1** Can only make simple meals (sandwiches/breakfast etc.).<br>**2** Reheats meals prepared by carer/meals on wheels/makes tea.<br>**3** Needs meals served to them / Unsafe (hazard) in kitchen.	
Housekeeping/ Laundry	N ☐ Y ☐<br>↓ →	**1** Assistance needed for heavy housework only (hoovering).<br>**2** Assistance needed for light housework (dishes, laundry).<br>**3** Unable to do any housework / laundry/ unsanitary conditions	
Transportation<br>(**Not** referring to driving ability)	N ☐ Y ☐<br>↓ →	**1** Can arrange own transport out of house (call taxi, lift).<br>**2** Needs someone to accompany them outside the house.<br>**3** Cannot travel outside house even with assistance/housebound.	
Medications	N ☐ Y ☐<br>↓ →	**1** Needs prompting to take medications./ needs meds organised.<br>**2** Needs to be given some (e.g. subcut insulin) /all medications.<br>**3** Poor compliance / inappropriate administration / refusing.	
Finances	N ☐ Y ☐<br>↓ →	**1** Directs people but cant manage complex banking.<br>**2** Needs assistance with bills, money, poor concept of value.<br>**3** Taken care of by other/no concept of money/ financial abuse.	
**C. Other**	N ☐ Y ☐	Specify ____________________<br>**1 2 3**	

Domain 3.	Step 1	Step 2		Step 3
**Issues**	**Concern**	**Status**		**Care Network**
**Medical State**	Is there concern about issues in this domain?(Circle Yes or No)	**Circle the present level of function**<br>**1. Mild.**<br>**2. Moderate.**<br>**3. Severe.**		Can the caregiver network manage the concern for this domain?<br>**1.Can manage**<br>**2.Carer strain**<br>**3.Some gaps**<br>**4.Cannot manage**<br>**5.Absent/liability**
	N ☐ Y ☐<br>↓ →	If NO concern, complete the Global Risk Score.<br>If YES complete each section below.		
**A. Med issues**	If NO concern, move on to next section until domain is complete.			
Chronic medical condition(s)<br>**Exclude mental state issues**	N ☐ Y ☐<br>↓ →	1<br>2<br>3	Asymptomatic /condition(s) controlled / no recent exacerbation.<br>Symptoms but not affecting function / recent exacerbation.<br>Frequent exacerbations / affecting function.	
Symptoms/ Palliative care issues (e.g.pain)	N ☐ Y ☐<br>↓ →	1<br>2<br>3	Mild chronic symptoms/terminal condition: asymptomatic or symptoms well controlled.<br>Ongoing symptoms needing specialist input.<br>Active symptoms (e.g. pain) ongoing despite specialist input/actively dying.	
**B. Physical**				
Hearing	N ☐ Y ☐<br>↓ →	1<br>2<br>3	Reduced hearing /uses hearing aid to help.<br>Difficulty hearing (+/- despite hearing aid).<br>Profoundly deaf, marked difficulty communicating.	
Vision	N ☐ Y ☐<br>↓ →	1<br>2<br>3	Reduced visual acuity but normal eyesight (wears glasses).<br>Visually impaired / (+/- despite glasses).<br>No vision and interfering with function.	1 2 3 4 5<br>↓
Communication	N ☐ Y ☐<br>↓ →	1<br>2<br>3	Expressive dysphasia, difficulty communicating but intelligible.<br>Mixed dysphasia, marked difficulty communicating.<br>Aphasic or non-communicating.	
Swallow	N ☐ Y ☐<br>↓ →	1<br>2<br>3	History / concern of aspiration but not evident at present.<br>Episodes of aspiration, needs diet modified.<br>Aspirating/non compliance with diet/ swallow absent /needs or using peg.	
Nutrition	N ☐ Y ☐<br>↓ →	1<br>2<br>3	History/ concern of malnutrition/ BMI upper/lower limits of norm.<br>Malnourished, abnormal BMI.<br>Evidence of serious malnutrition, severe anorexia or obesity.	
Gait / Falls	N ☐ Y ☐<br>↓ →	1<br>2<br>3	History of fall in the last year (none recently)/fear of falling/difficulty walking or with balance.<br>Abnormal gait pattern/recent falls.<br>Gait grossly abnormal/frequent falls/no safety awareness.	
Environment/ socioeconomics	N ☐ Y ☐<br>↓ →	1<br>2<br>3	Concern raised over home environment/ social isolation/disadvantaged area.<br>Poor sanitation or structural housing conditions/marked social isolation.<br>Dangerous sanitary, structural housing or social conditions.	
**C. Other**	**Y N**	Specify ____________________<br>1 2 3		

Global Risk Score

	1	2	3	4	5
**A.**<br>**Institutionalization**<br>Overall risk of admission to long-term care {nursing home} in the next year.	**Minimal**<br>**Rare**<br>☐	**Low**<br>**Unlikely**<br>☐	**Moderate**<br>**Possible**<br>☐	**High**<br>**Likely**<br>☐	**Extreme**<br>**Certain**<br>☐

	1	2	3	4	5
**B.**<br>**Hospitalization**<br>Risk of hospitalization including prolonged admission or readmission in the next year.	**Minimal**<br>**Rare**<br>☐	**Low**<br>**Unlikely**<br>☐	**Moderate**<br>**Possible**<br>☐	**High**<br>**Likely**<br>☐	**Extreme**<br>**Certain**<br>☐

	1	2	3	4	5
**C.**<br>**Death**<br>Risk of death in the next year.	**Minimal**<br>**Rare**<br>☐	**Low**<br>**Unlikely**<br>☐	**Moderate**<br>**Possible**<br>☐	**High**<br>**Likely**<br>☐	**Extreme**<br>**Certain**<br>☐

Comments:____

Signed:____________**Role/position:** ______**Years of experience:**___**Date: / /**
